# Effects of a Virtual Reality‐Based Dementia Educational Program on Healthcare Staff in Geriatric Dementia Wards: A Pre‐Post Comparative Study

**DOI:** 10.1111/psyg.70099

**Published:** 2025-09-22

**Authors:** Jinyan Wu, Ayumi Igarashi, Manami Takaoka, Haruna Kugai, Hiroshige Matsumoto, Noriko Yamamoto‐Mitani

**Affiliations:** ^1^ Department of Gerontological Home Care and Long‐Term Care Nursing, Division of Health Sciences & Nursing, Graduate School of Medicine The University of Tokyo Tokyo Japan; ^2^ Department of Advanced Gerontological Nursing, Graduate School of Nursing, School of Nursing Chiba University Chiba Japan; ^3^ Department of Nursing Data Science, Graduate School of Medicine University of Tokyo Tokyo Japan; ^4^ Department of Community Health Nursing/Public Health Nursing, Division of Health Sciences & Nursing, Graduate School of Medicine The University of Tokyo Tokyo Japan

**Keywords:** attitude, dementia, educational program, multidisciplinary, person‐centered care, virtual reality

## Abstract

**Background:**

Multidisciplinary collaboration is essential to the provision of effective and comprehensive support in dementia care. In Japan, Wards for Elderly Patients with Dementia (WEDs) prioritize team‐based care but face challenges due to insufficient staff training. This study developed a virtual reality‐based training program to enhance attitudes toward dementia and promote multidisciplinary collaboration among WEDs staff and evaluated its overall effectiveness.

**Methods:**

We designed a multidisciplinary workplace program based on input from WEDs staff, incorporating short films, virtual reality experiences, discussions, mini‐lectures, and a dilemma‐based case discussion. Staff from six WEDs in a hospital specializing in dementia care participated in the program. Pre‐ and post‐program questionnaires assessed the participants' attitudes and empathy toward people living with dementia and their awareness of multidisciplinary collaboration. Changes in person‐centered care practices were evaluated using a 1‐month follow‐up survey.

**Results:**

A total of 27 employees (10 nurses, 13 rehabilitation staff, 1 trainee doctor, 1 radiology technician, 1 nursing assistant, and 1 system official) completed the program. The mean age was 34.4 (±10.9) years, with 17 being female. After the program, significant improvements were observed in the participants' attitude toward people living with dementia (pre‐ vs. post‐evaluation: 43.4 vs. 45.1, respectively, *p* = 0.01), empathy (15.9 vs. 16.8, *p* = 0.01), and awareness of multidisciplinary collaboration (14.2 vs. 15.3, *p* = 0.005). Among the 14 participants who responded to the follow‐up survey, no significant change was observed in person‐centered care practices (28.1 vs. 28.4, *p* = 0.61).

**Conclusions:**

The virtual reality‐based dementia educational program improved attitudes, empathy, and awareness of multidisciplinary collaboration among WEDs staff, suggesting its potential as an innovative educational tool. Future research using larger and more diverse samples is warranted to validate its broader applicability and long‐term behavioral impact.

## 1 Introduction

The global increase in the number of people living with dementia (PLWD) has made the management of behavioral and psychological symptoms of dementia (BPSD) a crucial aspect of dementia care [1]. BPSD encompasses a range of symptoms including hallucinations, delusions, night‐time wandering, and aggressive behaviors, which significantly impact the quality of life of individuals with dementia and their caregivers [1, 2]. Effective management of these symptoms often requires a team‐based approach, with professionals from various fields working together to provide comprehensive care [3]. This multidisciplinary collaboration is particularly important during acute exacerbations or when physical complications make home care challenging. In such cases, temporary admission to specialized facilities, such as the Special Care Unit for Persons with Dementia and BPSD (SCU‐B), is recommended [4]. SCU‐Bs, distinct from general day care centers or special care units within nursing homes, focus on managing severe symptoms and supporting a return home. These units prioritize patient dignity and aim for short‐term stays rather than long‐term care.

In Japan, similar facilities exist in the form of Wards for Elderly patients with Dementia (WEDs), which provide structured care for patients with severe symptoms [5, 6]. WEDs are run by multidisciplinary teams, typically including a full‐time psychiatrist, experienced nurses, a full‐time occupational therapist (OT), and a full‐time psychiatric social worker (PSW). The establishment of WEDs has been internationally recognized as an effective approach to managing BPSD, providing a structured environment where both medical and psychosocial interventions can be administered [4, 7, 8].

However, despite the critical role of WEDs, significant challenges in the training and education of staff working in these wards persist. While recent studies highlight the increasing need for comprehensive dementia education targeting hospital staff and have developed numerous educational programs [9, 10], few address the unique educational needs of WEDs staff. A survey conducted with 18 nurses working in WEDs revealed that 47% experienced high workloads and fatigue, with 91% citing the lack of conferences and educational opportunities as a key concern [11]. A specialised educational program for nurses specialising in dementia care, including those working in WEDs, led to a significant increase in both knowledge‐based and practice‐based self‐efficacy among participants [12]; however, no multidisciplinary programs have been identified. Furthermore, non‐medical professionals, such as administrative staff and nursing assistants, who frequently interact with dementia patients, often receive minimal to no dementia care training [9].

Studies have shown that staff in specialised dementia care settings often struggle with BPSD management [13], leading to increased stress, decreased empathy, and occasionally negative attitudes toward patients [14]. Empathy‐based training programs have been recommended to reduce personal distress, prevent burnout, and improve the overall care environment [15]. Given these challenges, an educational program is needed to enhance staff empathy while minimising additional workload; such a program should be integrated easily into work routines and offer practical, experience‐based learning without imposing a heavy burden on staff.

In recent years, several studies have explored the use of virtual reality (VR) in dementia education for healthcare professionals, demonstrating its effectiveness in improving empathy, reducing stigma, and enhancing the understanding of PLWD [16, 17]. VR‐based training offers immersive learning experiences that allow participants to gain a deeper emotional and cognitive understanding of dementia [18]. Building on this evidence, our research team developed a VR‐based dementia education program designed to foster empathy toward PLWD. This program, applied to the general public [19], nursing students [20], and healthcare professionals [21], has shown improvements in attitudes toward PLWD and intentions of helping behavior. However, these previous programs were designed for single professional groups and did not incorporate interprofessional training elements, which are crucial in multidisciplinary settings like WEDs.

In this study, we adapted the VR program to reflect the training needs of WEDs staff and explored its feasibility and potential effects on empathy, attitudes, awareness of multidisciplinary collaboration, and person‐centered care (PCC) practices among multidisciplinary staff.

## 2 Methods

### 2.1 Design

This study employed a one‐group pre‐test and post‐test design. All participants were provided web‐based documents describing the study's aims, procedures, and voluntary nature of participation. The participants reviewed this information and indicated their consent by checking a box on the web form before beginning the program. The study received ethical approval from the first author's institutional ethics committee (2021359NI‐(3)).

### 2.2 Study Setting and Participants

The study took place at a hospital specializing in dementia care, which includes six WEDs. The participants were limited to hospital staff directly involved in dementia care, including doctors, nurses, nursing assistants, rehabilitation staff, medical technologists, and clerical staff. The inclusion criteria were being employed at the hospital and being proficient in Japanese. Recruitment was facilitated by the director of the nursing department, who distributed program leaflets to each department. Staff members could apply for participation voluntarily via an internet registration form.

### 2.3 Program Development Process

**FIGURE 1:**
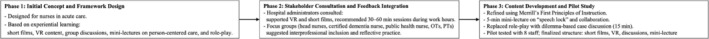
Development Process of the VR‐based Dementia Education Program. Note: VR, virtual reality.

The VR‐based dementia education program was developed in several phases, involving a collaborative approach with input from various stakeholders to ensure its relevance and effectiveness for staff working in WEDs (Figure 1).

#### 2.3.1 Initial Concept and Framework Design

The original program for healthcare professionals was designed for nurses in acute care hospitals [21]. The design was guided by experiential learning principles, emphasizing both theoretical knowledge and practical application through interactive activities like role‐playing and scenario‐based discussions. It combined short films and VR content, depicting the perspective of an older woman with dementia, followed by group discussions and a role‐play to reflect on care approaches. Mini lectures on PCC were also included to reinforce key concepts [21].

#### 2.3.2 Stakeholder Consultation and Feedback Integration

A key aspect of the program development was gathering feedback from healthcare professionals at various stages. Firstly, a consultation session was held with hospital administrators to review the program concept, and all attendees responded positively to the VR and short film components. They also recommended conducting the training sessions during working hours to encourage participation and suggested an optimal duration of 30 to 45 min, with a maximum of 1 h.

Subsequent focus group interviews were conducted with eight healthcare professionals from different disciplines, including three head nurses, one certified dementia nurse, one public health nurse, two OTs, and one physiotherapist. These interviews highlighted specific challenges in dementia care, such as the limited opportunities for staff to reflect on their practices and the need for more interprofessional collaboration. Requests to include non‐medical staff, such as administrative workers, in the training to enhance team understanding were also made. These insights were incorporated into the final program design.

#### 2.3.3 Content Development and Pilot Study

In light of the identified challenges and needs of the training, a tailored program was designed following Merrill's First Principles of Instruction, which focused on creating effective and efficient learning experiences by actively engaging learners in meaningful tasks [22]. The program maintained the core structure of short films, VR films, and discussion sessions, while the lecture component was condensed to 5 min, focusing specifically on “speech lock” and interprofessional collaboration. Instead of an original role‐play, a 15‐min group‐based discussion using realistic dementia care dilemmas was introduced under the supervision of a certified dementia care nurse. The entire program was designed to fit within a 50‐min session.

After conducting a pilot study with eight staff members (including nurses, nursing assistants, and rehabilitation staff), the final structure of the program was confirmed. It included short films, VR videos, discussions, a focused mini‐lecture, and a dilemma‐based case discussion (Table 1).

**TABLE 1:** Structure of the VR‐based dementia education program.

Merrill's first principles of instruction	Purpose	Contents	Time duration
Problem‐centered	Engage learners in solving real‐world problems	**1. Short film (Undesirable scenario)**	8 min
		Viewing a story about a female PLWD, participants contemplate her background and the responses of the surrounding people.	
Activation	Recall and apply prior knowledge	**2. VR experience film (Undesirable scenario)**	5 min
		Participants experience a female PLWD's perspective and share her emotions and the responses of the surrounding people.	
		**3. Discussion**	5 min
		Participants share their impressions after the undesirable VR and short films, and consider how they would respond to PLWD.	
Demonstration	Show examples rather than just telling	**4. VR experience film (Desirable scenario)**	5 min
		Participants learn good responses and perceive a positive emotional change in PLWD through a comparison between the two scenarios.	
		**5. Short film (Desirable scenario)**	7 min
		Participants identify the differences in PLWDs' emotions and the surrounding people's responses between the two scenarios.	
		**6. Lecture**	5 min
		Emphasising avoiding “Speech Lock” and the need for multidisciplinary collaboration.	
Application	Use new knowledge in a practical way	**7. Dilemma‐Based Case Discussion**	15 min
		Discussing real‐world care dilemmas based on the that the short film's main character is hospitalised, exploring support strategies in interprofessional groups.	
Integration	Reflect and apply knowledge in practice	Follow‐up survey on changes in practice after one month provides opportunity for reflection	

*Note:* Contents 1–5 are from the original program; contents 6 and 7 were newly developed.

Abbreviations: PLWD, people living with dementia; Speech Lock, restricting or controlling someone's actions through words; VR, virtual reality.

### 2.4 Newly Developed Contents

#### 2.4.1 Short Films, VR, and Discussion

**FIGURE 2:**
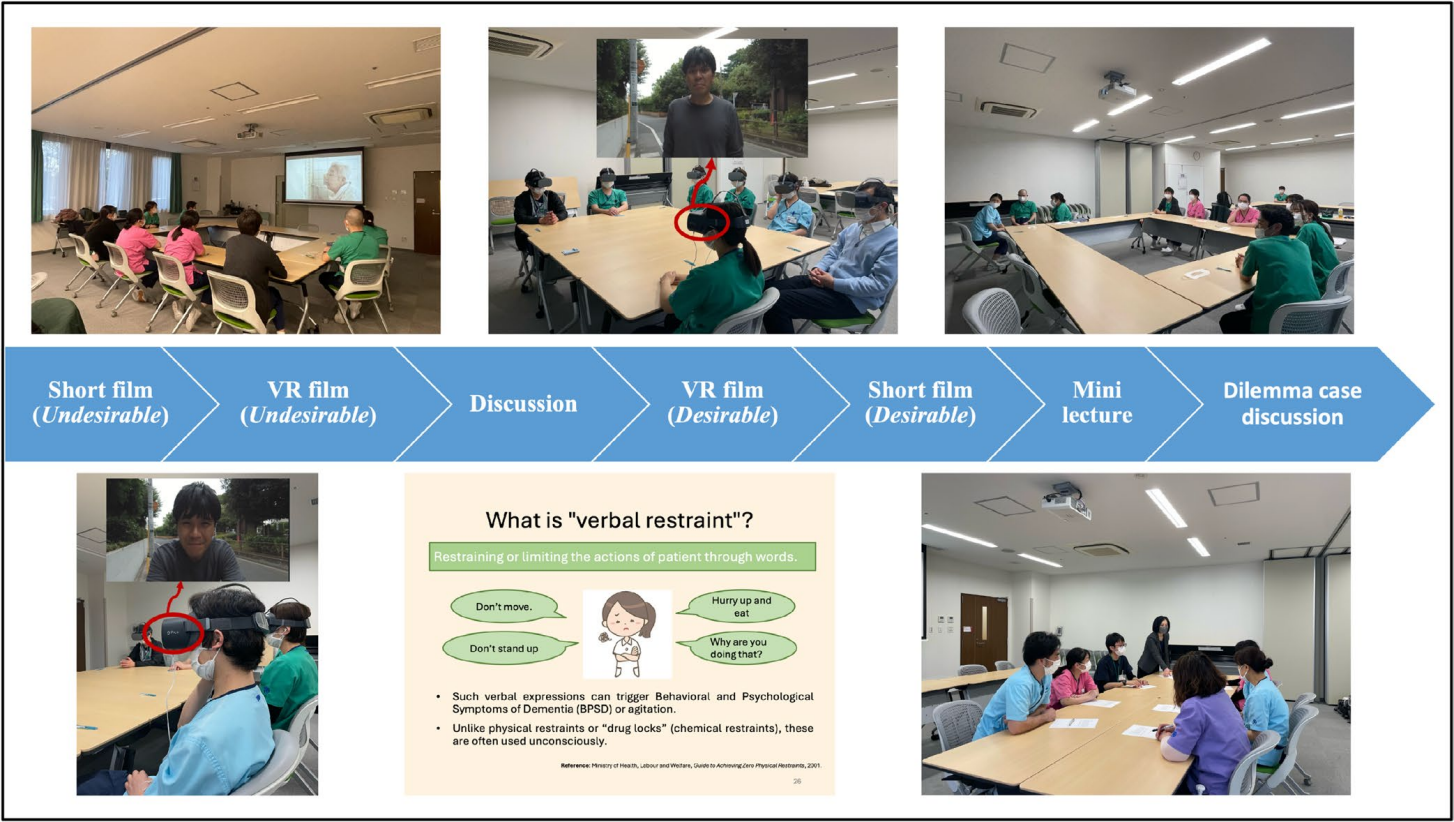
Implementation of the VR‐based Dementia Education Program. Note: VR, virtual reality.

To engage participants emotionally, short films and VR scenarios depicting both “desirable” and “undesirable” approaches to dementia care were used (Figure 2). This allowed participants to experience the impact of interacting with patients and encouraged reflection on their own practices. After viewing the undesirable short films and VR, the participants engaged in discussions to reflect on their experiences and consider how they would apply these insights in their own care settings. For further details on these elements, please refer to our earlier publication [21], where these methods are thoroughly explained. Details of the VR scenarios are provided in Appendix S1.

#### 2.4.2 Mini‐Lecture

A review of previous studies [23] and preliminary interviews with staff revealed that although the staff working in WEDs possess knowledge about dementia, they face challenges related to “speech lock” and multidisciplinary collaboration in dementia care. “Speech lock” refers to the act of verbally restricting or controlling a person's behavior, which is said to cause BPSD and disquiet. Therefore, the lecture emphasized the importance of avoiding “speech locks” and fostering teamwork and collaboration among different professionals involved in dementia care.

#### 2.4.3 Dilemma‐Based Case Discussion

Based on feedback from the interviews, we found that the original role‐play segment for acute care hospitals did not fully address the unique challenges faced in WEDs. In response, we developed a scenario‐based session that focused on real‐world dilemmas in dementia care, tailored to this specialized environment. The purpose was to create realistic scenarios that reflected the actual difficulties encountered by different professional roles, providing a safe space for participants to discuss and strategize solutions collaboratively.

The dilemma cases, designed under the supervision of a certified dementia nurse, were based on the perspectives of nurses, rehabilitation staff, and general administrative staff, as these roles accounted for the majority of the target participants. Each scenario posed challenging decisions without a clear answer, prompting the participants to weigh complex considerations and reflect on their practice. The participants assumed that the female protagonist in the VR content and the short film had been admitted to their hospital, and this helped them ground the scenarios in their work context. For instance, the scenario from the perspective of the rehabilitation staff was as follows: “You visit a patient for rehabilitation and find the patient deeply asleep. The nurse asks you to wake the patient for rehabilitation, despite the patient's sleep‐wake cycle being reversed. What would you do?” To facilitate decision‐making, the participants were presented with two options: YES or NO.

The participants were divided into interprofessional groups of 4 to 5 members, allowing them to approach these dilemmas from diverse professional perspectives. Each group selected one of three dilemma cases, discussed their proposed actions, chose YES or NO, and shared their reasoning. The discussions also included strategies for addressing these challenges, encouraging participants to reflect on their own practices and collaborate across disciplines.

### 2.5 Structure Based on Merrill's First Principles

The program was implemented in accordance with the sequence of activities shown in Table 1. This structured flow ensured that each session was dynamic, engaging, and focused on practical application, ultimately promoting empathetic and effective dementia care within a multidisciplinary context.

#### 2.5.1 Problem‐Centered Approach

To root the program in real‐world challenges, the participants were introduced to the perspective of an older woman with dementia through a short film showing an “undesirable” scenario. This approach set the foundation for experiencing empathy, as the participants were encouraged to consider the woman's background and the responses of those around her while reflecting on issues commonly encountered in dementia care.

#### 2.5.2 Activation Phase

Building on their prior experiences, participants then engaged with a VR simulation of the same “undesirable” scenario, allowing them to further immerse themselves in the patient's perspective and experience the emotional responses of those around her. This activity, combined with a group discussion, prompted participants to draw on their past dementia care experiences and consider improvements in their own interactions.

#### 2.5.3 Demonstration Phase

The participants then viewed a “desirable” scenario in both short film and VR formats, showcasing effective practices. The contrast between the scenarios helped them identify positive care approaches, particularly in terms of the emotional well‐being of PLWD and caregiver reactions. This phase included a concise mini lecture on “speech lock” and the importance of multidisciplinary collaboration, illustrating key dementia care practices and reinforcing positive caregiver interactions.

#### 2.5.4 Application Phase

The participants then engaged in a scenario‐based session, applying their new knowledge to real‐world dilemma cases specific to WEDs. In interprofessional groups, they discussed potential actions in scenarios without clear answers, fostering collaboration and shared problem‐solving within diverse perspectives.

#### 2.5.5 Integration Phase

To reinforce long‐term improvements in dementia care practices, the participants were encouraged to reflect on their learning through a follow‐up survey conducted 1 month after the program. Although this follow‐up was not part of the intervention itself, it aligned with the Integration Phase of Merrill's First Principles of Instruction, as it provided the participants with an opportunity to evaluate how they applied their insights in daily practice.

The program was conducted four times in February 2024, with 6–9 participants per session, and was scheduled during work hours to encourage attendance. To accommodate staff availability and scheduling constraints, the sessions were organized in a cross‐ward format, allowing participants from different wards to attend together. Sessions were held in the hospital meeting room (Figure 2) and facilitated by the first author and other study members.

### 2.6 Outcome Measures

The program's effectiveness was evaluated using the participants' attitudes toward PLWD as the primary outcome, with empathy, awareness of multidisciplinary collaboration, and PCC as secondary outcomes. Attitude was prioritized as it influences key aspects of dementia care, including empathy and PCC practices. We hypothesized that VR's first‐person experience, supplemented by short films, enhances empathy and fosters a positive attitude toward PLWD. In turn, this attitude shift was expected to improve the participants' understanding of other professional roles and promote multidisciplinary collaboration. The lecture and scenario‐based discussion aimed to reinforce these changes by deepening role awareness and fostering collaborative approaches in dementia care. The participants completed the following questionnaires before and after participating in the program. Attitudes toward PLWD, empathy, and awareness of multidisciplinary collaboration were assessed immediately before and after the program, whereas changes in PCC practices were assessed one month later via a follow‐up survey.

#### 2.6.1 Primary Outcomes

##### 2.6.1.1 Attitudes Toward PLWD

Participants' attitudes were assessed using the Attitudes Toward Dementia Scale [24], a validated 14‐item scale rated on a four‐point Likert scale: 1 = disagree, 2 = mostly disagree, 3 = mostly agree, 4 = agree. This scale has four subscales: tolerance (five items), refusal (four items), feeling of distance (three items), and affinity (two items). It has demonstrated good psychometric properties (Cronbach's α = 0.60–0.81). Example items include, “I can share joy and pleasure with people with dementia” and “As much as possible, I do not want to engage with people with dementia.” Scores were reversed for negatively worded items, and the total score was calculated by summing the 14 items, with scores ranging from 14 to 56. Higher scores indicated a more positive attitude.

#### 2.6.2 Secondary Outcomes

##### 2.6.2.1 Empathy

Empathy was measured using the Empathic Support Behaviour Scale for Nurses [25], a validated 14‐item scale rated on a 5‐point Likert scale (from strongly disagree to strongly agree). This scale has two factors with high internal consistency. Our study specifically focused on the “Sense of Responsibility” subscale, which includes four items with a total score of 1–20, with higher scores indicating greater empathy. Cronbach's alpha for this subscale was 0.80. An example item is, “When I see a patient (client) in distress, I can't help but reach out to them.”

##### 2.6.2.2 Awareness of Multidisciplinary Collaboration

Awareness of multidisciplinary collaboration was assessed using four self‐designed items based on an existing scale on interprofessional collaboration in community care [26]. No validated scale was identified that specifically captured day‐to‐day interprofessional awareness within dementia care wards. Therefore, we developed a concise 4‐item scale (score range: 4–20) tailored to the ward‐based dementia care context, while minimizing respondent burden. The items were designed to promote reflection on the importance of multidisciplinary collaboration, particularly in terms of understanding the roles and perspectives of other professionals and sharing dementia care challenges. Items were rated on a five‐point Likert scale (1 = strongly disagree to 5 = strongly agree), with higher scores indicating stronger collaboration awareness.

An example item is, “I understand the perspectives and care strategies of other professionals working with the same patient (or client).” The full list of items is provided in Appendix S2.

##### 2.6.2.3 Person‐Centered Care Practices

PCC was evaluated using seven self‐developed items adapted from a validated self‐assessment scale originally aimed at nurses caring for older adults with cognitive impairment [27]. The items were modified to suit the multidisciplinary WEDs setting, and a simplified 7‐item version was created to reduce response burden. Participants responded based on their usual care practices, not a specific time frame. Each item was rated on a five‐point Likert scale (1 = strongly disagree, 5 = strongly agree), with a total score range of 7 to 35. Higher scores indicated more person‐centered care.

An example item is, “Understand behavior from the patient's perspective first when responding to BPSD.” The full list of items is provided in the results section.

### 2.7 Data Analysis

We used paired *t*‐tests to compare changes in outcomes from pre‐test to post‐test and from pre‐test to 1‐month post‐test, with effect sizes estimated using Cohen's d. To check the robustness of our results, we conducted subgroup analyses focusing on age (< 30 vs. ≥ 30), occupation (nurse vs. rehabilitation staff), and years of experience (< 5 years vs. ≥ 5 years), as these factors were expected to influence the outcomes. A two‐tailed *p*‐value < 0.05 was considered statistically significant. Statistical analyses were performed using IBM SPSS Statistics version 29.0 (IBM Corp., Armonk, NY, USA).

## 3 Results

### 3.1 Participant Characteristics

Approximately 200 staff members across the hospital were informed of the program. A total of 52 registered for participation, 29 of whom attended the session. Two participants were excluded from the analysis due to prior participation in the pilot study, resulting in a final analytic sample of 27 participants (10 nurses, 13 rehabilitation staff, 1 trainee doctor, 1 radiology technician, 1 nursing assistant, and 1 system official). The main reasons for non‐attendance were COVID‐19 and seasonal influenza outbreaks within the hospital.

Fourteen participants (51.8%) responded to the follow‐up survey. The mean age of the participants was 34.4 (±10.9) years, with 63.0% being female. Notably, 29.6% had no prior experience with dementia training (Table 2).

**TABLE 2:** Participants' demographic and professional characteristics.

		*n* = 27	
		*n* (%)/Mean ± SD	
Age	Total	34.4 ± 10.9	
	20s	13	48.2
	30s–50s	13	48.2
Sex (female)		17	63.0
Occupation	Nurses	10 (37.0)	37.0
	Nursing assistant	1 (3.7)	3.7
	Rehabilitation staff	13 (48.2)	48.2
	Trainee doctor	1 (3.7)	3.7
	Radiology technician	1 (3.7)	3.7
	Systems official	1 (3.7)	3.7
Staff		24	88.9
Management		3	11.1
Highest educational qualification	Vocational course	17	63.0
	University	10	37.0
Years of experience		6.3 ± 6.6	
	Nurses	9.4	8.0
	Rehabilitation staff	2.8	2.2
No prior experience with dementia training		8	29.6

*Note:* Missing data were excluded from each item. Percentages were calculated based on valid responses.

Abbreviations: PLWD, people living with dementia; SD, standard deviation.

### 3.2 Changes in Attitudes, Empathy, Multidisciplinary Collaboration, and PCC Practices

Significant improvements were observed in the participants' attitudes toward PLWD, empathy, and multidisciplinary collaboration after the program (Table 3). While overall PCC practices did not exhibit significant changes, one specific item showed notable improvement.

- **Attitudes Toward PLWD:** The total score increased significantly from 43.4 at pre‐evaluation to 45.1 at post‐evaluation (*p* = 0.01). Among the subscale, “Tolerance” increased significantly from 16.9 to 17.5 (*p* = 0.03), while “Refusal” (10.8 to 11.4, *p* = 0.10), “Feeling of distance” (9.1 to 9.3, *p* = 0.62), and “Affinity” (6.7 to 7.0, *p* = 0.06) did not show statistically significant changes.
- **Empathy:** The total score increased significantly from 15.9 at pre‐evaluation to 16.8 at post‐evaluation (*p* = 0.01).
- **Awareness of Multidisciplinary Collaboration:** The total score increased significantly from 14.2 at pre‐evaluation to 15.3 at post‐evaluation (*p* = 0.005).
- **PCC Practices:** The overall score remained stable, increasing slightly from 28.1 at pre‐evaluation to 28.4 at post‐evaluation (*p* = 0.61). However, one item, “Try to be receptive to the patient's unique needs and anxieties,” showed a significant improvement, with the mean scores increasing from 4.1 at pre‐evaluation to 4.4 at 1‐month post‐evaluation (*p* = 0.04).

**TABLE 3:** Pre‐ and post‐intervention changes in outcome measures.

	Mean ± SD (*n* = 27)							
	Pre			Post			*p* ^a^	*d* ^b^
Attitude (Total score)	43.4	±	3.5	45.1	±	3.8	0.**012**	**0.54**
Tolerance	16.9	±	1.7	17.5	±	1.8	0.**033**	**0.45**
Refusal	10.8	±	1.5	11.4	±	1.6	0.100	0.33
Feeling of distance	9.1	±	1.9	9.3	±	1.8	0.621	0.10
Affinity	6.7	±	0.8	7.0	±	1.0	0.059	0.38
Empathy	15.9	±	2.3	16.8	±	1.9	0.**012**	**0.52**
Multidisciplinary collaboration	14.2	±	2.8	15.3	±	2.6	0.**005**	**0.60**
PCC Practices (Total score)	28.1	±	2.8	28.4	±	3.2	0.606	0.14
1. Encourage and support from the perspective of “what can be done” rather than “what cannot.”	4.0	±	0.6	4.1	±	0.5	0.547	0.17
2. Understand behaviour from the patient's perspective first when responding to BPSD.	4.1	±	0.7	4.2	±	0.6	0.336	0.27
3. Try to be receptive to the patient's unique needs and anxieties.	4.1	±	0.5	4.4	±	0.5	0.**040**	**0.61**
4. Respond politely and courteously when the patient makes unreasonable demands.	3.8	±	0.6	3.7	±	0.9	0.752	0.09
5. Make an effort to compensate for memory loss by explaining place and time.	3.8	±	0.7	3.9	±	0.9	0.500	0.19
6. Communicate clearly with words or gestures.	4.3	±	0.7	4.1	±	1.1	0.547	0.17
7. Explain and gain agreement on what to do next from the patient's perspective.	4.1	±	0.6	3.9	±	0.7	0.435	0.22

Abbreviation: SD, standard deviation.

^a^ Bold values indicate statistically significant results at *p* < 0.05.

^b^ Effect sizes (Cohen's d) were calculated based on paired *t*‐tests. Interpretation: small (0.00–0.17), moderate (0.18–0.43), large (0.44–0.84).

In the subgroup analysis (Table 4), participants with ≤ 5 years of experience showed significant improvements in attitude (*p* = 0.03, d = 0.65) and empathy (*p* = 0.003, d = 0.92), while those with > 5 years of experience showed no statistically significant changes in these outcomes. Participants in their 20s showed significant improvement in empathy (*p* = 0.001, d = 1.25), as did rehabilitation staff (*p* < 0.001, d = 1.44). In contrast, no significant changes were observed in empathy among participants aged ≥ 30 years or among nurses. Nurses showed significant improvements in awareness of multidisciplinary collaboration (*p* = 0.02, d = 0.92). Additionally, rehabilitation staff also showed a significant improvement in perceived multidisciplinary collaboration (*p* = 0.04, d = 0.63).

**TABLE 4:** Stratified pre‐ and post‐intervention changes in three outcomes by participant characteristics.

		Attitude				Empathy				Multidisciplinary collaboration			
		Mean ± SD				Mean ± SD				Mean ± SD			
		Pre	Post	*p* ^a^	*g* ^b^	Pre	Post	*p* ^a^	*g* ^b^	Pre	Post	*p* ^a^	*g* ^b^
Experience	0–5 years (*n* = 15)	43.1 (3.2)	45.4 (3.2)	0.**026**	0.65	16.1 (2.3)	17.4 (2.0)	0.**003**	0.92	14.1 (2.7)	14.8 (3.0)	0.061	0.53
	> 5 years (*n* = 12)	43.9 (4.0)	44.7 (4.6)	0.280	0.36	15.6 (2.2)	16.0 (1.7)	0.499	0.20	14.4 (3.1)	15.8 (1.9)	0.**040**	0.67
Age	20s (*n* = 13)	43.7 (3.8)	45.8 (4.2)	0.059	0.61	16.9 (1.9)	18.1 (2.0)	0.**001**	1.25	14.1 (2.6)	15.3 (2.6)	0.**032**	0.71
	> 30 years (*n* = 13)	43.6 (3.4)	44.3 (4.1)	0.396	0.27	15.3 (2.1)	15.7 (1.0)	0.468	0.21	14.7 (3.1)	15.6 (1.9)	0.125	0.46
Occupation	Rehabilitation (*n* = 13)	42.7 (2.9)	44.4 (3.0)	0.119	0.47	16.2 (2.4)	17.2 (2.4)	**< 0.001**	1.44	14.1 (3.1)	14.9 (3.1)	0.**043**	0.63
	Nursing (*n* = 10)	45.6 (2.9)	46.6 (4.3)	0.256	0.41	15.9 (0.7)	16.6 (1.6)	0.132	0.52	15.1 (1.4)	16.4 (6.3)	0.**018**	0.92

Abbreviation: SD, standard deviation.

^a^ Bold values indicate statistically significant results at *p* < 0.05.

^b^ Effect sizes (Cohen's d) were calculated based on paired *t*‐tests. Interpretation: small (0.00–0.17), moderate (0.18–0.43), large (0.44–0.84).

## 4 Discussion

This study aimed to develop and evaluate a workplace dementia education program using VR and scenario‐based discussion, tailored for staff working in WEDs. The results demonstrated significant improvements in attitudes toward PLWD, empathy, and awareness of multidisciplinary collaboration, highlighting the potential value of this program in addressing both emotional and team‐based aspects of dementia care. To our knowledge, this is the first study to implement a VR‐based dementia education program for WEDs staff, incorporating a multidisciplinary training component.

Nearly one‐third of the participants had no prior dementia training despite working in a specialised ward, indicating a critical need for ongoing education even in settings that might be presumed to have sufficient expertise. The program, designed in accordance with Merrill's First Principles, may offer a feasible and impactful model to address this gap. The program's activation and demonstration phases—delivered via short films and VR—allowed the participants to emotionally connect with PLWD and challenge their preconceptions. Previous studies have shown that storytelling interventions, such as films illustrating patients' life histories, can effectively humanize patients and improve care providers' attitudes by enhancing emotional engagement and empathy [28]. Likewise, immersive VR experiences have been demonstrated to significantly improve empathy by enabling healthcare providers to vividly understand the emotional and cognitive experiences of PLWD [16, 29]. Consistent with these findings, our study observed significant improvements in attitudes and empathy, indicating the potential usefulness of combining storytelling and VR to reinforce emotional engagement and positive attitude shifts [30].

The application phase, involving scenario‐based dilemma discussions, encouraged reflection and peer discussion across professional roles. While the participants initially considered the dilemmas from their assigned professional role, the discussions naturally evolved to incorporate their individual perspectives, prior experiences, and personal insights. Prior research highlights that multidisciplinary education, particularly interactive case‐based discussions, can enhance healthcare professionals' awareness of team‐based approaches and improve collaborative practice skills [31]. In line with this finding, our results indicate that structured, interactive discussions may have effectively facilitated the participants' understanding of their colleagues' roles, reinforcing multidisciplinary collaboration and team cohesion in dementia care. The VR and video components, which preceded the application phase, may have played an important role in fostering this collaborative mindset. By providing an immersive, experiential understanding of PLWD's perspectives, these components likely increased participants' empathy and sensitivity toward person‐centred needs. This heightened awareness may have encouraged participants to engage more meaningfully in the dilemma‐based discussions, enabling them to better appreciate diverse professional viewpoints and approach the scenarios with a shared, person‐centred focus.

Subgroup analysis revealed nuanced findings. Younger participants and those with less experience showed larger improvements in empathy and attitudes. This pattern may reflect the stronger responsiveness of early‐career professionals to VR‐based experiential learning, which tends to align more closely with the learning styles and digital fluency of younger generations [18, 32]. Rehabilitation staff also demonstrated significant gains in empathy. Their roles often involve prolonged, one‐on‐one engagement with patients in emotionally dynamic settings, which may have made the emotional content of the program particularly salient and relevant to their daily practice. However, as nearly half the sample were rehabilitation staff, the observed significant changes may partly reflect their larger representation, which increased the statistical power to detect differences in this subgroup.

In contrast, nurses showed limited improvement in empathy, possibly explained by emotional fatigue or desensitisation from prolonged exposure to complex care (mean experience = 9.4 years), as reported in previous studies [13, 14]. Nevertheless, they demonstrated the largest gains in awareness of multidisciplinary collaboration following the training. Rehabilitation professionals also showed significant improvements, albeit with smaller effect sizes than nurses. This pattern may suggest that structured case‐based discussion is particularly beneficial for experienced professionals—especially nurses—in facilitating critical reflection on team‐based roles.

Although the program was designed to include non‐medical professionals, their participation was extremely limited due to scheduling and other logistical barriers. This limits the generalizability of our findings on multidisciplinary collaboration, as perspectives from staff such as administrative workers and nursing assistants were underrepresented. Given their frequent contact with PLWD, their involvement is important. Future studies should consider flexible formats, such as asynchronous or on‐demand training, to enhance their participation.

Despite improvements in attitudes and empathy, no substantial changes were observed in actual PCC‐related care behaviours. Limited follow‐up survey responses may have influenced our ability to detect such changes. However, one item—“Try to be receptive to patients' unique needs and anxieties”—showed a significant improvement. Although categorised under care behaviours, this item may reflect an attitudinal shift, suggesting a potential early sign of behavioural change. While the program appears to have influenced participants' perspectives on dementia care, translating such gains into consistent practice often requires more than a single training event. Behavioural change in clinical settings is a complex and gradual process, requiring sustained reinforcement, peer support, and institutional facilitation. To bridge this gap, future interventions could incorporate follow‐up coaching, structured reflection sessions, and team‐based PCC action plans to support the application of PCC principles in daily practice [33].

This study has several limitations. The small sample size, absence of a control group, and limited inclusion of non‐medical staff restrict the generalizability of the findings. The COVID‐19 pandemic significantly impacted recruitment, restricting participation and limiting participant diversity. The lack of a control group prevented us from isolating the program's effects from external influences, such as workplace culture or pre‐existing motivation among participants. Furthermore, a short follow‐up period and reliance on self‐reported measures may have also contributed to this limitation. Future research should involve larger, more diverse samples, include control groups, and adopt longitudinal designs to evaluate the sustained impact of such programs on dementia care.

## 5 Conclusions

The VR‐based dementia education program was associated with improvements in WEDs staff's attitudes toward PLWD, empathy, and awareness of multidisciplinary collaboration. Although no significant changes were observed in PCC practices, the findings highlight the potential value of a structured educational program—incorporating VR and interactive discussion elements—as a practical and engaging approach to dementia training. Future studies should refine the program structure, extend follow‐up periods, and examine its impact across diverse roles and settings to support sustainable improvements in dementia care.

## Disclosure

The authors have nothing to report.

## Conflicts of Interest

The authors declare no conflicts of interest.

## Supporting information


**Appendix S1:** VR scenario contents.
**Appendix S2:** Items used to assess awareness of multidisciplinary collaboration.

## Data Availability

The data that support the findings of this study are available on request from the corresponding author. The data are not publicly available due to privacy or ethical restrictions.
