# Supplementary material for: Effects of a Virtual Reality‐Based Dementia Educational Program on Healthcare Staff in Geriatric Dementia Wards: A Pre‐Post Comparative Study
Source: Psychogeriatrics. 2025 Sep 22;25(6):e70099. doi: 10.1111/psyg.70099 (PMC12453941; doi:10.1111/psyg.70099)
Supplement: Supplementary file 1 — Appendix S1: VR scenario contents. Appendix S2: Items used to assess awareness of multidisciplinary collaboration. [file PSYG-25-0-s001.docx]

| **Appendix 1**. VR Scenario Contents |
| --- |
|  |
| Two 5-min VR films were shown, each composed of five scenes filmed from a first-person perspective of an older female person living with dementia (PLWD). |
| For each scene, two versions were created to contrast “desirable” and “undesirable” communication styles, helping participants reflect on person-centred care. In each scene, participants experienced the PLWD’s perspective and heard her inner thoughts. |
| The five scenes were: |
| **1. Having tea with friends at a café** – experiencing memory loss and difficulty following the conversation. |
| **2. Unable to open the restroom door** – illustrating impaired judgment. |
| **3. Meeting a neighbor on the street** – forgetting the name of an acquaintance. |
| **4. Getting lost while returning home** – depicting disorientation. |
| **5. Reuniting with her son after getting lost** – showing the emotional impact of disorientation. |

Note. VR, virtual reality

**Appendix 2.** Items Used to Assess Awareness of Multidisciplinary Collaboration

| 1. I can contact other professionals involved in the same patient (or client) without hesitation. |  |  |
| --- | --- | --- |
| 2. I generally understand the difficulties faced by professionals from other disciplines involved in the patient’s (or client’s) care. | | |
| 3. I understand the perspectives and care strategies of other professionals working with the same patient (or client). | | |
| 4. I have opportunities to share and discuss challenges related to dementia care with other professionals. | |  |

- Participants rated the following four items on a 5-point Likert scale (1 = strongly disagree, 5 = strongly agree).
- The total score ranged from 4 to 20, with higher scores indicating greater awareness of multidisciplinary collaboration.
